# Supplementary material for: A One-Year Systematic Study to Assess the Microbiological Profile in Oysters from a Commercial Harvesting Area in Portugal
Source: Microorganisms. 2023 Jan 29;11(2):338. doi: 10.3390/microorganisms11020338 (PMC9965842; doi:10.3390/microorganisms11020338)
Supplement: Supplementary file 1 [file microorganisms-11-00338-s001.zip › microorganisms-2161578-supplementary.pdf]

Supplementary Material:

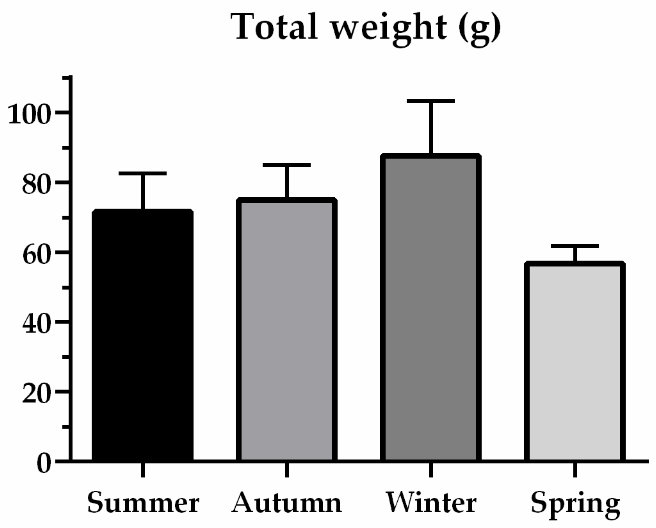

(a)

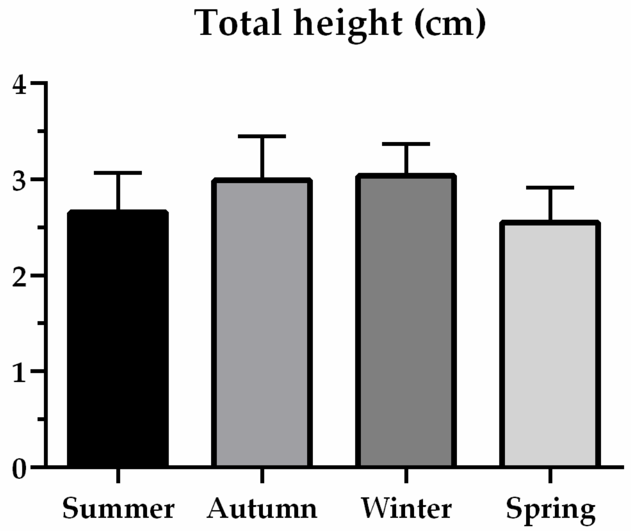

(b)

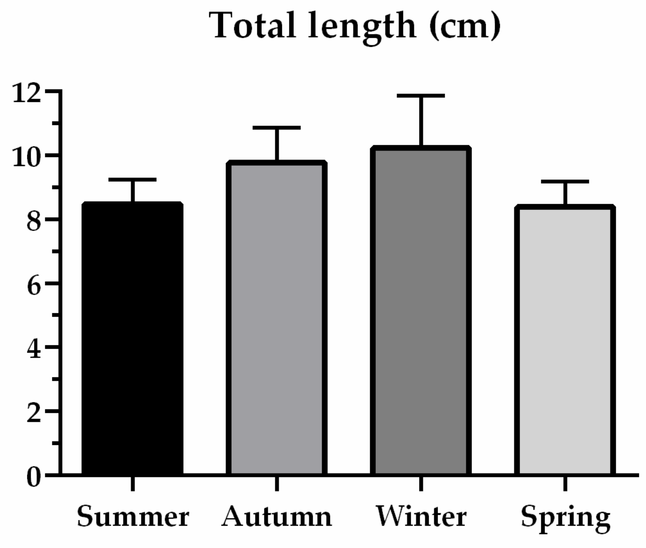

(c)

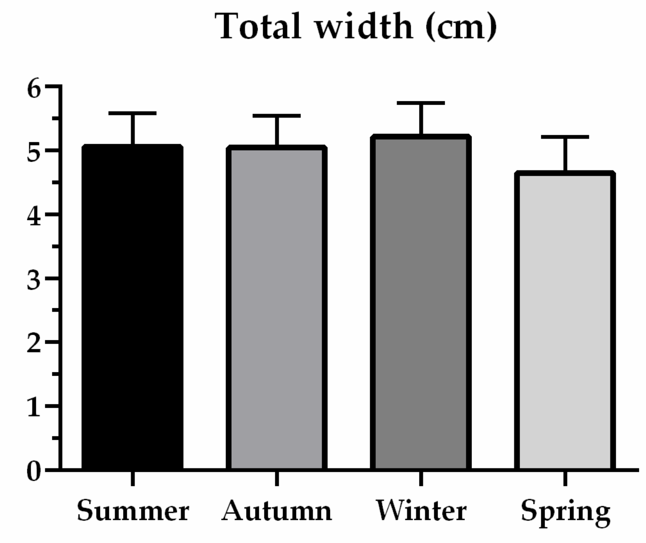

(d)

Figure S1. (**a**) Total weight variation in grams; (**b**) total height variation in centimeters, (**c**) total length variation in centimeters; (**d**) total width variation in centimeters of oysters during each season.

Table S1. Summary of the methodology used for bacteriologic analysis.

Microorganism	Method	Revitalization period			Selective period		
		Medium	T (°C)	duration	Medium	T (°C)	duration
Total aerobic microorganism	ISO 4833-1<br>ISO 6222	BPW [1,2]<br>NA [3]	Room temperature [1,2]<br>NA [3]	1h [1,2]<br>NA [3]	PCA	7 [1]<br>22 [3]<br>30 [1,2]<br>37 [3]	72h<br>72h<br>72h<br>48h

Marine heterotrophic bacteria	ILM	BPW [1,2] NA [3]	Room temperature [1,2] NA [3]	1h [1,2] NA [3]	MA	21	48h
E. coli	ISO 16649-2 ISO 16649-3	BPW	Room temperature	1h	TBX	44	24h
Salmonella spp.	ISO 6579	MSRV	41,5	24h	XLD	37	24h
C. perfringens	ISO 7937	NA	NA	NA	TSC	37	20h
Coagulase-positive *Staphylococcus*	ISO 6888-3	BPW	Room temperature	1h	BP	37	48h
Enterococcus spp.	ISO 7899-2	BPW	Room temperature	1h	SB	37	48h
L. monocytogenes	ISO 11290-1	Half-Fraser broth	37	24h	Compass® Listeria	37	24h
Yeasts and molds	ISO 21527-2	BPW	Room temperature	1h	Sab	25	5 to 7 days
Pseudomonas spp.	ILM	BPW	Room temperature	1h	CFC	30	48h

[1] Edible portion; [2] Intra-valvular liquid, superficial biofilm and hemolymph, [3] Farming water column samples, BP: Baird-Parker medium, BPW: Buffered Peptone Water, CFC: Cephaloridine Fucidin Cetrimide medium, ILM: internal laboratory method, MA: Marine Agar medium, MSRV: Modified Semi-solid Rappaport-Vassiliadis, NA: not applicable, PCA: Plate Count Agar medium, Sab: Sabourau medium, SB: Slanetz and Bartley medium, TSC: Tryptose Sulfite Cycloserine agar, XLD: Xylose Lysine Deoxycholate agar.

Table S2. Summary of morphological parameters throughout the seasons.

Parameter	Season	Average	SD	Max	Min	95% CI		p-value
						L	U	
Total weight	Summer	71.6	10.9	103.0	55.7	66.7	76.6	****
	Autumn	75.0	9.9	88.10	48.7	71.0	79.0	
	Winter	84.3	18.5	131.7	65.9	81.2	94.1	
	Spring	56.9	5.0	67.0	49.0	55.1	58.6	
Total height	Summer	2.7	0.4	3.3	1.9	2.5	2.8	****
	Autumn	3.0	0.5	4.0	2.1	2.8	3.2	
	Winter	3.0	0.3	3.9	2.6	2.9	3.2	
	Spring	2.6	0.4	3.5	1.6	2.4	2.7	
Total length	Summer	8.4	0.8	9.6	7.3	8.1	8.8	****
	Autumn	9.8	1.1	11.7	7.5	9.3	10.2	
	Winter	10.2	1.6	12.7	3.6	9.6	10.9	
	Spring	8.4	0.8	9.8	7.2	8.1	8.7	
Total width	Summer	5.1	0.5	6.1	3.9	4.8	5.3	***
	Autumn	5.0	0.5	6.0	4.1	4.8	5.2	
	Winter	5.2	0.5	6.2	4.0	5.0	5.4	
	Spring	4.7	0.6	6.1	3.6	4.4	4.8	

SD: standard deviation, Max: maximum, Min: minimum, CI: confidence interval, L—Lower limit; U—Upper limit, * $p \leq 0.05$, ** $p \leq 0.01$, *** $p \leq 0.001$, **** $p \leq 0.0001$ *p*-value was calculated by one-way ANOVA.
